# Short-Term Outcomes of Pediatric Patients With Mild Autosomal Recessive *RPE65*-Associated Retinal Dystrophy Treated With Voretigene Neparvovec

**DOI:** 10.1167/tvst.14.8.8

**Published:** 2025-08-04

**Authors:** David A. Merle, Leen Hertens, Spyridon Dimopoulos, Susanne Kohl, Manon Van Haute, Elfride De Baere, Marieke De Bruyne, Barbara Janssens, Klaus Rüther, Cord Huchzermeyer, Pascale Mazzola, Fanny Nerinckx, Tobias Haack, Lasse Wolfram, Melanie Kempf, Laura Kühlewein, Krunoslav Stingl, Bart P. Leroy, Katarina Stingl

**Affiliations:** 1 Department of Ophthalmology, University Eye Clinic, Eberhard Karls University of Tübingen, Tübingen, Germany; 2 Institute for Ophthalmic Research, Department for Ophthalmology, Eberhard Karls University of Tübingen, Tübingen, Germany; 3 Department of Ophthalmology, Medical University of Graz, Graz, Austria; 4 Department of Ophthalmology, Ghent University Hospital, Ghent, Belgium; 5 Center for Medical Genetics Ghent, Ghent University Hospital, Ghent, Belgium; 6 Department of Biomolecular Medicine, Ghent University, Ghent, Belgium; 7 Ophthalmological Practice Hippocrates, Sint-Niklaas, Belgium; 8 Ophthalmological Practice Berlin-Mitte, Berlin, Germany; 9 Department of Ophthalmology, University Hospital Erlangen, Friedrich-Alexander-University Erlangen-Nürnberg, Erlangen, Germany; 10 Institute for Medical Genetics and Applied Genomics, University Hospital, Eberhard Karls University Tübingen, Tübingen, Germany; 11 Center for Rare Eye Diseases, Eberhard Karls University of Tübingen, Tübingen, Germany; 12 Department of Head and Skin, Ghent University, Ghent, Belgium

**Keywords:** RPE65, gene therapy, retina, voretigene neparvovec

## Abstract

**Purpose:**

Voretigene neparvovec is approved for *RPE65*-associated inherited retinal degeneration (*RPE65*-IRD) in the United States and the European Union. According to current knowledge, early treatment benefits efficacy. However, consensus on treating mild cases is lacking due to ambiguity in balancing clinical benefits with potential side effects. Therefore, we present short-term outcomes of four pediatric patients with milder types of *RPE65*-IRD.

**Methods:**

Two unrelated pediatric patients were unilaterally treated at the University Eye Hospital in Tübingen, Germany. Another two unrelated pediatric patients were bilaterally treated at Ghent University Hospital, Belgium. Examinations were performed before and until at least 6 months after treatment, including best-corrected visual acuity, slit-lamp examination, fundus photography, short-wavelength fundus autofluorescence, optical coherence tomography, 90° kinetic perimetry, dark-adapted chromatic perimetry, and full-field stimulus threshold measurements.

**Results:**

Despite surgical challenges, treatment with voretigene neparvovec was successful in all four patients. All patients showed rod functional rescue with stable best-corrected visual acuity. Three patients suffered chorioretinal atrophy at the retinotomy site but none developed signs of fast-growing CRA. One case developed limited CRA in the bleb area, potentially related to inflammation in the subretinal space.

**Conclusions:**

Treatment with voretigene neparvovec was safe and effective in patients with mild *RPE65*-IRD. Early treatment showed good functional outcomes. Also, treatment at stages without profound retinal degeneration might lower the risk of fast-growing CRA.

**Translational Relevance:**

This study aids clinical decision-making in unclear cases by demonstrating that early treatment with voretigene neparvovec in mild *RPE65*-IRD provides functional benefits while minimizing the risk of fast-growing chorioretinal atrophy.

## Introduction

Retinoid isomerohydrolase (RPE65) plays a pivotal role in the visual cycle, and functional impairments are known to cause severe forms of inherited retinal degenerations (IRDs), such as Leber congenital amaurosis 2 (LCA2; OMIM#204100) and retinitis pigmentosa (RP; OMIM#613791 and OMIM#618697). Due to either complete lack of or reduced retinoid isomerase function of the RPE65 protein, affected individuals experience a shortage of 11-*cis*-retinal, a vitamin A derivative that serves as a light-absorbing component of the visual pigments in photoreceptors and enables phototransduction in the photoreceptors. Due to this lack of 11-*cis*-retinal, various degrees of night blindness are typically among the earliest complaints, and most cases show rapid progression of retinal degeneration, with a large proportion of patients becoming legally blind in early adulthood and many cases progressing to complete blindness thereafter. Despite rare cases of autosomal dominant inheritance,^1^^–^^3^ *RPE65*-associated IRDs typically follow an autosomal recessive mode of inheritance.^4^^,^^5^ Although *RPE65*-associated IRDs are generally regarded as fast progressing, there is an increasing number of reports on very mild phenotypes, frequently associated with potentially hypomorphic *RPE65* variants.^6^^–^^9^ It is acknowledged that there is no universally accepted definition of a mild phenotype. However, clinicians often encounter cases where the decision between watchful waiting and initiating therapy is challenging. These cases frequently involve patients who initially present with very good visual acuity or relatively complete visual fields, as observed in our patients. Such cases are not typically representative of the standard *RPE65*-IRD phenotype.

Voretigene neparvovec, a subretinally injected gene therapy for autosomal recessive *RPE65*-IRD, was approved by the U.S. Food and Drug Administration in 2017 and by the European Medicines Agency in 2018 based on a seminal phase 3 clinical trial that demonstrated improved functional vision among participants based on multi-luminance mobility test (MLMT) performance.^10^ Individuals 3 years old and above with confirmed biallelic *RPE65* disease-associated variants were considered eligible if their visual acuity was 20/60 or poorer and/or their visual field was narrowed to less than 20° in any meridian while still displaying a sufficient number of viable and therefore treatable retinal cells.^10^ In the following years, multiple post-marketing studies were able to show improved or at least stable visual function after treatment, confirming the safety and efficacy data of the phase 3 trial.^11^^,^^12^

Despite these positive reports, some patients treated with voretigene neparvovec develop patches of fast-growing chorioretinal atrophy (CRA) over time. Although some patients only suffer from atrophic areas at the retinotomy site, potentially caused by the elevated mechanical pressure, flow, and/or local toxicity during the subretinal injection, some patients develop fast-growing patches of CRA after the application of voretigene neparvovec, usually located in the retinal midperiphery. These atrophic patches often lie outside of the surgical bleb area, do not always resemble the typical phenotype seen during the natural course of autosomal recessive *RPE65*-IRD, and are not associated with retinotomy sites.^13^ Increasing clinical evidence shows that, although an immunological etiology cannot be excluded, these different types of CRA might be a consequence of increased metabolic turnover after photoreceptor rescue in a degenerated retina with elevated RPE65 function induced by voretigene neparvovec treatment.^14^^–^^16^ Detailed multimodal retinal diagnostics have shown that areas with the greatest increase of rod function are often the initial sites of atrophy growth.^16^ To date, it cannot be predicted preoperatively which patients are at risk of developing such lesions with absolute certainty or how those lesions will progress in the long term, adding some degree of uncertainty to an otherwise very promising therapeutic avenue. It has been reported repeatedly that the younger age at treatment, the higher the chance of treatment success.^13^ However, published research indicates that pediatric patients, who typically experience significant benefit from treatment, are more susceptible to the development of CRA.^15^ Remarkably, very young children are usually not affected by this adverse effect.

In clinical routine, devoid of the clearly defined criteria of clinical trials, clinicians face significant clinical heterogeneity in the phenotypes of patients presenting with *RPE65*-associated dystrophies. Although advanced cases with some degree of residual visual function are readily assumed to benefit from the treatment, mild cases are significantly more difficult to decide on. On the one hand, for very advanced cases with only a few remaining functional outer retinal cells, a more limited treatment effect is expected,^11^^,^^15^ whereas the procedure-associated risks and the economic implications remain. On the other hand, decision-making for milder phenotypes in pediatric patients presents a significant challenge. Opting not to treat or deferring intervention until there is evidence of more substantial progression could be advantageous in the short term, as it spares children surgery-associated risks, particularly when the potential for improved retinal function is minimal in very mild cases. Conversely, early intervention can yield greater long-term benefits, as a less degenerate retina is more capable of efficiently integrating enhanced functionality compared to one with more advanced degeneration. Additionally, development of CRA is mostly observed in teenage years and young adulthood, but to a lesser extent in younger children.^15^

Unfortunately, data on treatment outcomes in such mild phenotypes, especially in children, are scarce, making decisions about to treat or not to treat exceedingly difficult. Therefore, we report the clinical outcomes of four mild pediatric cases of *RPE65*-associated retinal dystrophy that have been successfully treated with voretigene neparvovec at the University Eye Hospital Tübingen, Germany, and at Ghent University Hospital, Belgium.

## Methods

### Patients

Two patients, a 6-year-old boy and a 12-year-old girl, were clinically examined and unilaterally treated at the University Eye Hospital Tübingen, and two patients, a 12-year-old girl and a 6-year-old-girl, were clinically examined and bilaterally treated at Ghent University Hospital.

### Ethics and Informed Consent

This study was conducted in accordance with the tenets of the Declaration of Helsinki. Ethical approval was obtained from the ethic committees at the University of Tübingen and Ghent University Hospital. Verbal and written consent was obtained from the parents for the use of the patients’ clinical and genetic data for research purposes, in agreement with German and Belgian legislation.

### Clinical Examinations

Patients were followed up at regular time points prior to and after treatment, and comprehensive ophthalmological examinations were performed at initial presentation to the centers before surgery (baseline) and at 3 and 6 months after surgery. The examinations included the following:

• Best-corrected visual acuity (BCVA) using Early Treatment Diabetic Retinopathy Study (ETDRS) logMAR letter and tumbling E charts
• Slit-lamp and dilated fundus examinations
• Color fundus photography (CFP)—Tübingen, with a ZEISS Clarus 700 (Carl Zeiss Meditec, Oberkochen, Germany) and California P200DTx (Optos, Dunfermline, UK); Ghent, with a TRC-50IX (Topcon, Tokyo, Japan) and ZEISS Clarus 700
• Fundus autofluorescence (FAF)—Tübingen, with a California P200DTx; Ghent, with a SPECTRALIS device (Heidelberg Engineering, Heidelberg, Germany)
• Optical coherence tomography (OCT)—Tübingen, with SPECTRALIS; Ghent, with SPECTRALIS OCT Plus
• 90° kinetic perimetry, Goldmann visual field (GVF)—Tübingen, with Octopus 900 (Haag-Streit, Köniz, Switzerland); Ghent, with a manual Goldmann perimeter
• Dark-adapted chromatic perimetry (DACP), used only for case 2 (Medmont International Pty Ltd, Nunawading, Vic, Australia) using a grid of 36 test points with a stimulus size of 1.73° (Goldmann size V) with 0 dB = 17.6 cd/m^2^

Dark-adapted thresholds were measured either with the full-field stimulus threshold (FST) test using blue and red lights with 0 dB set to 0.01 cd/m^2^ (at Tübingen, Espion E2 and E3; Diagnosys, Lowell, MA) or with the full-field dark adaptation protocol using blue, red, and white lights (at Ghent, Espion units), without previous bleaching. For an improved comparability of the findings, all FST values reported here are also provided as decimal logarithmic values of the absolute luminance (lg cd/m^2^), and dark-adapted thresholds are provided in decibels (assuming 0 dB corresponded to 0.01 cd/m^2^).

With the above-mentioned settings for FST, normal values for dark-adapted, rod-mediated FST using a blue stimulus are around −60 dB; for red, around −35 dB. In a retina where rods are still able to achieve a better dark-adapted sensitivity than cones, red and blue thresholds are mediated by rods, and a difference of at least 10 dB between blue and red values is indicative of these rod-mediated thresholds. Mixed cone-mediated thresholds are characterized by a difference between red and blue values lower than 10 dB.^17^^,^^18^ The FST (Tübingen) utilizes a forced-choice (yes/no) algorithm, where subjects respond via a two-button box. This results in a sigmoid curve (psychometric function). The perceptional threshold is defined as the stimulus luminance at the inflection point of the curve and is reported here in decibels (relative to 0.01 cd/m^2^). In contrast, the dark adaptation protocol (Ghent) uses a one-button box and the subject indicates stimulus detection. Here, the absolute thresholds were defined as the minimal stimulus luminance values that were perceived by the subject. These values were calculated as the average of 10 to 12 values over 5 to 6 minutes per measurement and are reported as dark adaptation values in log cd/m^2^. Both FST and dark adaptation measurements have been performed monocularly in mydriasis.

### Genetic Testing

For case 1, genetic diagnostic testing and segregation analysis were conducted in a commercial diagnostic genetic laboratory by comprehensive IRD panel sequencing on genomic DNA extracted from buccal swaps at the patient's age of 4 years. Two heterozygous missense variants in *RPE65* were detected, and segregation in both parents confirmed a biallelic *trans* configuration. For case 2, genetic diagnostic testing and segregation analysis were conducted on genomic DNA extracted from whole venous blood samples at the patient's age of 9 years at the Institute for Medical Genetics and Applied Genomics (IMGAG) Tübingen applying IRD gene panel analysis based on whole-genome sequencing.^19^ Two variants in *RPE65* were detected, and segregation in both parents confirmed a biallelic *trans* configuration. For cases 3 and 4, genetic diagnostic and parental segregation analyses were performed at the Center for Medical Genetics Ghent (CMGG) at Ghent University Hospital. Single gene (*RPE65*) and gene panel (RetNet v4) analyses were performed on genomic DNA extracted from whole venous blood samples at the patients’ ages of 4 and 2.5 years for cases 3 and 4, respectively. In both cases, two variants in *RPE65* were detected, and parental segregation confirmed a biallelic *trans* configuration.

Variant nomenclature is in accordance with Human Genome Variation Society recommendations^20^ and based on GenBank accession numbers NM_000329.3 and NP_000320.1, as well as Ensembl reference ENST00000262340.6 corresponding to the MANE Select transcript, with nucleotide 1 being the first nucleotide of the translation initiation codon ATG. Variant classification was performed according to the guidelines by the American College for Medical Genetics (ACMG).

### Subretinal Injection of Voretigene Neparvovec

#### University Eye Hospital Tübingen

The surgical procedure was performed according to the manual provided by the manufacturer and the German Society for Ophthalmology. A standard 23G pars plana vitrectomy was performed, and detachment of the posterior hyaloid was confirmed by injection of triamcinolone where deemed appropriate by the surgeon. A 41G cannula was used to place the retinotomy along the superior arcade, injecting 300 µL vector solution in the subretinal space targeting the macula with a foot pedal–controlled injection system.

#### Department of Ophthalmology Ghent

Treatment for both Belgian patients was bilateral but always performed consecutively with 1 or 2 weeks between eyes. Administration of 300 µL voretigene neparvovec was performed under general anesthesia by means of a single subretinal injection into the subretinal space after a pars plana vitrectomy using the EVA platform (DORC Global, Zuidland, The Netherlands). Posterior vitreous detachment was induced using the vitrectome, followed by triamcinolone injection and detachment of the posterior hyaloid using a 27G inner limiting membrane forceps. To prevent retinal detachment, peripheral 360° laser photocoagulation was performed. A 38G needle was used for subretinal injection with the retinotomy located along the superior arcades.

### Perioperative Management

#### University Eye Hospital Tübingen

Starting 3 days before surgery, an anti-inflammatory regimen was given for 7 days consisting of the equivalent of 1 mg/kg/day prednisolone with a maximum of 40 mg/day. Subsequently, the dose was tapered over 10 days.

#### Department of Ophthalmology Ghent

An anti-inflammatory regimen was started 3 days before surgery that consisted of 0.8 mg/kg/day with a maximum of 32 mg methylprednisolone (equivalent of 1 mg/kg/day of prednisolone) for either 23 or 30 days (interocular interval of 1 week or 2 weeks, respectively).

## Results

### Case 1

**Figure 1.**
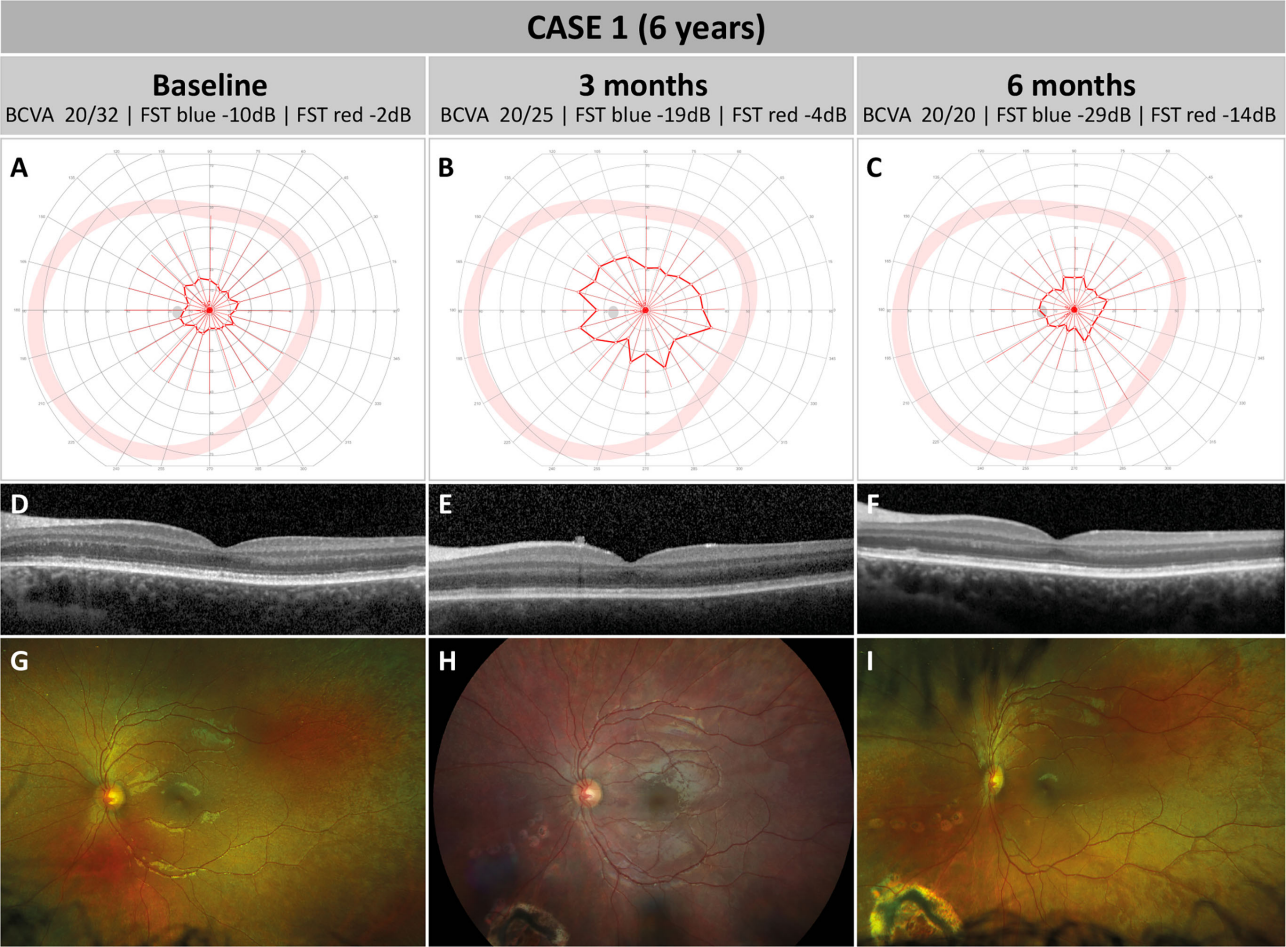
Pre- and post-treatment follow-up for case 1. The *left column* presents findings immediately prior to surgery (baseline). The *middle* and *right columns* show findings 3 months and 6 months after surgery, respectively. (**A**–**C**) Left-eye kinetic 90° perimetry using Goldmann object III4e. At all time-points, a concentric constriction to approximately 10° to 15° was measured, which was considered stable despite some variations in the measurement. *Solid red lines* represent the borders of the measured visual field; the *broader, pale red lines* represent reference values of healthy eyes. (**D**–**F**) Macular OCT scan of the left eye showed an irregular appearance of the EZ nasally at baseline, with a seemingly decreased granular appearance of the EZ at 3 and 6 months relative to baseline that was not explained by differences in image quality. A small hyperreflective accumulation superior to the retinal nerve fiber layer was detected at 3 months but was not detectable thereafter. (**G**–**I**) CFP at baseline and at 3 and 6 months after surgery. All pictures show a diffuse retinal atrophy typical for *RPE65*-associated IRD without spicular intraretinal pigment migration. The inferonasal retinal hole surrounded by laser scars along with some individual laser scars can be seen in panels (**H**) and (**I**). Limited, non-progressive CRA can be identified at the retinotomy site around the inferior vascular arcade in panels (**H**) and (**I**).

At the initial presentation to the University Eye Hospital Tübingen, this 4-year-old boy showed a BCVA of 20/20 in both eyes. Previous genetic analysis in an external lab found two compound heterozygous missense variants in *RPE65*—c.272G>A; p.(Arg91Gln) and c.560G>A; p.(Gly187Glu)—both classified as pathogenic (ACMG class 5). Both variants have repeatedly been reported in patients with *RPE65*-related retinal disease.^10^^,^^21^^,^^22^ No other variants in any known IRD-related genes were identified. Due to the young age of the patient, only a few examinations were possible. During the first visit, the opportunity for treatment with voretigene neparvovec was discussed with the parents, and it was jointly decided to continue to observe the patient before initiating the treatment. Two years later, at the age of 6, the patient presented to a follow-up examination (baseline). BCVA had decreased slightly to 20/32 in both eyes, and kinetic visual field testing using object III4e revealed an irregular concentric constriction to approximately 10° to 15° in both eyes (Fig. 1A; only left eye is shown). Left-eye FST measurements showed pathologically increased dark-adapted thresholds. Whether these were rod driven remains questionable, as indicated by FST values of −10 dB (−3 lg cd/m^2^) and −2 dB (−2.2 lg cd/m^2^) for blue and red light, respectively. On OCT, the outer retinal layers appeared to be relatively intact, although the ellipsoid zone (EZ) showed a subnormal, granular configuration (Fig. 1D). Fundoscopy revealed diffuse retinal atrophy, and CFP of the left eye is shown in Figure 1G. FAF images could not be captured due to limited patient cooperation and a diminished signal intensity that is typical for *RPE65*-associated IRDs.^23^^,^^24^ Based on the disease stage and by then increased clinical experience with voretigene neparvovec, the parents agreed to treat the patient's left eye as the first eye.

During vitrectomy, detachment of the posterior vitreous was difficult due to strong retinal adhesions and resulted in formation of a mid-peripheral retinal hole that required perilesional laser coagulation (Figs. 1H, 1I). Subretinal injection and bleb formation around the superior vascular arcade was complicated by strong adhesion of the foveal region with the retinal pigment epithelium. In order to detach the fovea, another bleb was induced at the lower vascular arcade; however, it was impossible to completely detach the foveal region. Other than these complications, surgery and postoperative management were uneventful.

Three months after surgery, the patient presented with a BCVA of 20/25 in the treated left eye which further increased to 20/20 at 6 months. Intriguingly, the FST values in the left eye improved to −19 dB (−3.9 lg cd/m^2^) and −4 dB (−2.4 lg cd/m^2^) at 3 months and to −29 dB (−4.9 lg cd/m^2^) and −14 dB (−3.4 lg cd/m^2^) at 6 months for blue and red light, respectively. The difference between the blue and red thresholds of 15 dB indicates a rod-mediated dark-adapted threshold and shows restoration of rod functionality. Kinetic perimetry at 3 months revealed an irregular concentric constriction to approximately 20° to 30° (Fig. 1B) that was interpreted as a possibly stable result, given the considerable variation observed in visual field testing prior to surgery. Furthermore, at the 6-month follow-up, the visual fields remained stable (Fig. 1C). Likewise, the macular OCT revealed a near-normal configuration of the retinal layers at 3- and 6-month follow-up (Figs. 1D–F). A small hyperreflective accumulation internal to the retinal nerve fiber layer was observed at 3 months (Fig. 1E) but was not detectable thereafter (Fig. 1F). On fundoscopy and the volume OCT scan, irregularities of the retinal surface/inner limiting membrane were visible after vitrectomy and a potential inflammatory etiology cannot be excluded. The previously noted granular appearance of the EZ seemingly decreased over time, giving rise to a more solid configuration as observable in healthy eyes. The patient reported subjectively improved vision under dim light conditions, accompanied by a slight increase in photophobia. Demographics and genetic variants of all cases are shown in Table 1, and longitudinal diagnostic results of all cases are shown in Table 2.

**Table 1.** Demographics and *RPE65* Variants

Case Number	Sex	Age at Treatment	*RPE65* Variant	ACMG Class
1	Male	6 y	c.272G>A; p.(Arg91Gln)	5
			c.560G>A; p.(Gly187Glu)	5
2	Female	12 y	c.200T>A; p.(Leu67Gln)	4
			c.1004A>G; p.(Glu335Gly)	3^*^
3	Female	12 y	c.11+5G>A	5
			c.991_993dupTGG (p.Trp331dup)	5
4	Female	6 y	c.271C>T, p.(Arg91Trp)	4
			c.1103A>G, p.(Tyr368Cys)	4

* Diagnosis of *RPE65*-associated IRD was based on clinical findings and the absence of other pathogenic variants in other IRD-associated genes.

**Table 2.** Longitudinal BCVA and FST Data

Case Number (Age at Treatment)	Treated Eye	Diagnostic Test	0 Months (Baseline)	3 Months	6 Months
Case 1 (6 y)	Left	BCVA	20/32	20/25	20/20
		FST blue (dB)	−10	−19	−29
		FST red (dB)	−2	−4	−14
Case 2 (12 y)	Left	BCVA	20/50	20/50	20/40
		FST blue (dB)	−20	−30	−32
		FST red (dB)	−5	−12	−17
Case 3 (12 y)	Right	BCVA	20/80	20/160	20/63
		FST blue (dB)	15	4	−14
		FST red (dB)	14	0	−2
		FST white (dB)	18	2	−3
	Left	BCVA	20/40	20/50	20/40
		FST blue (dB)	15	−4	−14
		FST red (dB)	13	3	0
		FST white (dB)	17	1	−6
Case 4 (6 y)	Right	BCVA	20/125	20/80	20/100^*^
		FST blue (dB)	12	−2	−29^†^
		FST red (dB)	22	4	−6^†^
		FST white (dB)	13	−1	−25^†^
	Left	BCVA	20/80	20/50	20/125^*^
		FST blue (dB)	10	−24	−38^†^
		FST red (dB)	25	0	−21^†^
		FST white (dB)	14	−16	−38^†^

* BCVA values are compromised in reliability due to suboptimal patient cooperation.

† FST measurements at the 6-month follow-up were unreliable; therefore, 12-month follow-up data are shown.

### Case 2

**Figure 2.**
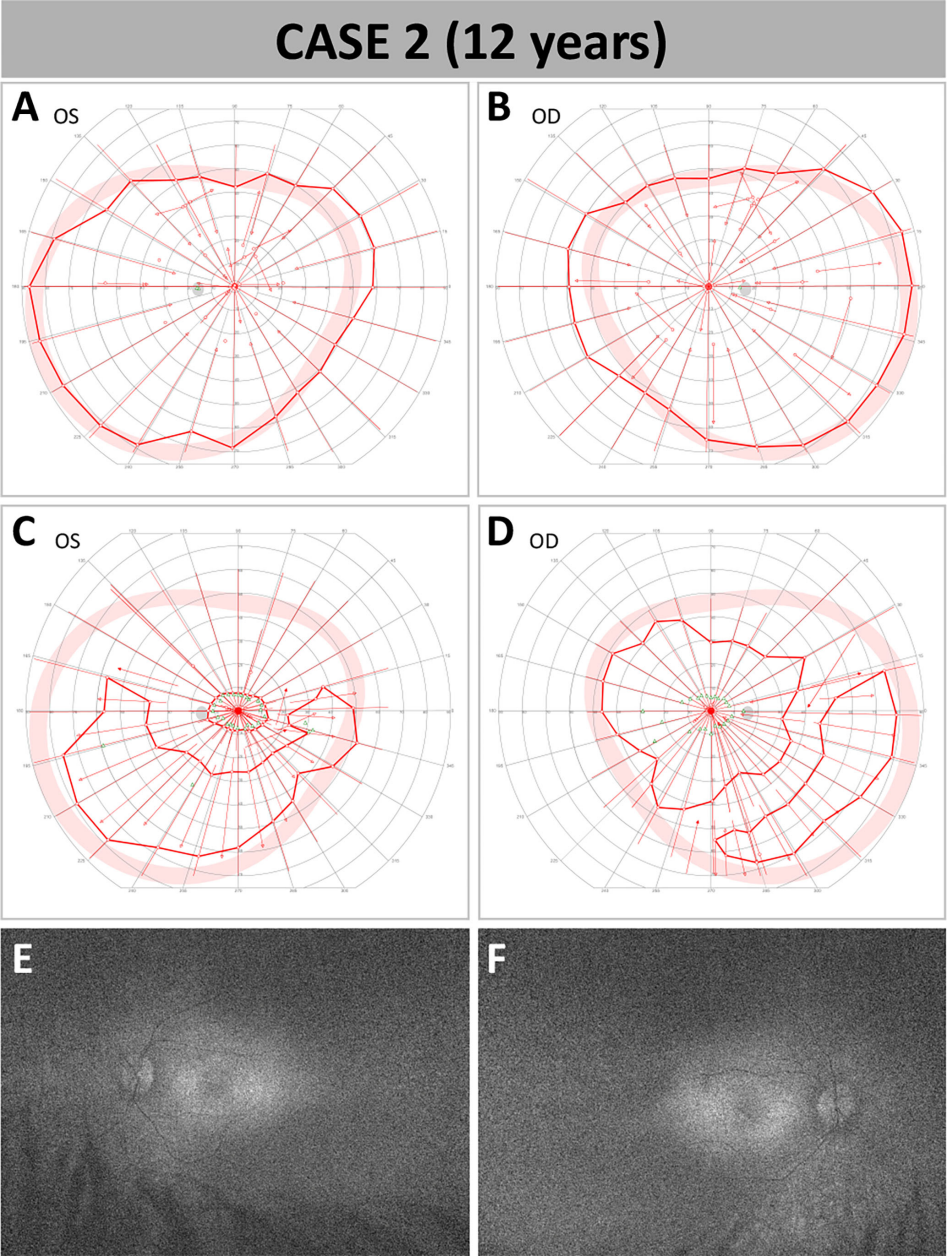
Initial presentation and pre-treatment follow-up of case 2. (**A**–**D**) The 90° Goldmann kinetic visual fields using object III4e showed clinically significant deterioration in both eyes within 3 years after initial presentation to our clinic: left eye at initial presentation (**A**), right eye at initial presentation (**B**), left eye at 3-year follow-up (baseline) (**C**), right eye at 3-year follow-up (baseline) (**D**). (**E**, **F**) FAF images of both eyes at baseline showing globally reduced but visible diffuse hyperautofluorescence at the posterior pole: left eye (**E**), right eye (**F**).

At initial presentation in our clinic, the girl was 9 years old and had a BCVA of 20/40 in the right eye and 20/63 in the left eye. Kinetic visual field testing using object III4e revealed near-normal visual fields in both eyes (Figs. 2A, 2B) and FST measurements showed reduced, albeit relatively well-preserved rod function in the left eye, with −40 dB (−6 lg cd/m^2^) and −16 dB (−3.6 lg cd/m^2^) for blue and red light, respectively. The retinal imaging showed a markedly reduced, although recordable, FAF signal, typical for the RPE65 phenotype (Fig. 2). Due to the automatic adaptation of the FAF signal, the average FAF value of the imaging was increased, resulting in a not-dark optic nerve in the images, characteristic of RPE65-associated dystrophy along with the reduced FAF intensity. Whole genome sequencing revealed two compound heterozygous missense variants in *RPE65*—c.200T>A; p.(Leu67Gln) and c.1004A>G; p.(Glu335Gly). Both variants are novel and unique to this patient. They have never been observed in patients nor in population databases (i.e., gnomAD, in-house database).^25^ They affect highly conserved amino acid residues, but prediction software scores were inconsistent for c.1004A>G; p.(Glu335Gly) but uniformly deleterious for c.200T>A; p.(Leu67Gln). For c.1004A>G; p.(Glu335Gly), another missense variant affecting the same amino acid residue has been reported once in ClinVar.^26^ For c.200T>A; p.(Leu67Gln), another missense variant at the same codon has been reported repeatedly in the literature and ClinVar in causal relationship with *RPE65*-related retinal disease.^27^ Both variants were initially classified as variants of uncertain significance. Of note, single heterozygous variants in *USH2A*—c.11864G>A; p.(Trp3955Ter)—and in *RP1L1*—c.5788G>T; p.(Glu1930Ter)—were also observed in the patient. Biallelic configuration of the *RPE65* variants was confirmed via parental segregation analysis. Based on the typical clinical findings, including very weak signal in FAF, a hallmark of only *LRAT*-IRD and *RPE65*-IRD,^23^^,^^24^ and in the absence of any other potentially causative genetic alterations, a diagnosis of autosomal recessive *RPE65*-IRD was made. Genetic reassessment led to the upgrade of the variant c.200T>A; p.(Leu67Gln) to likely pathogenic (ACMG class 4). Yet, based on the very mild phenotype and the scarcity of outcome data at that time, a joint decision was made to observe and not to treat.

**Figure 3.**
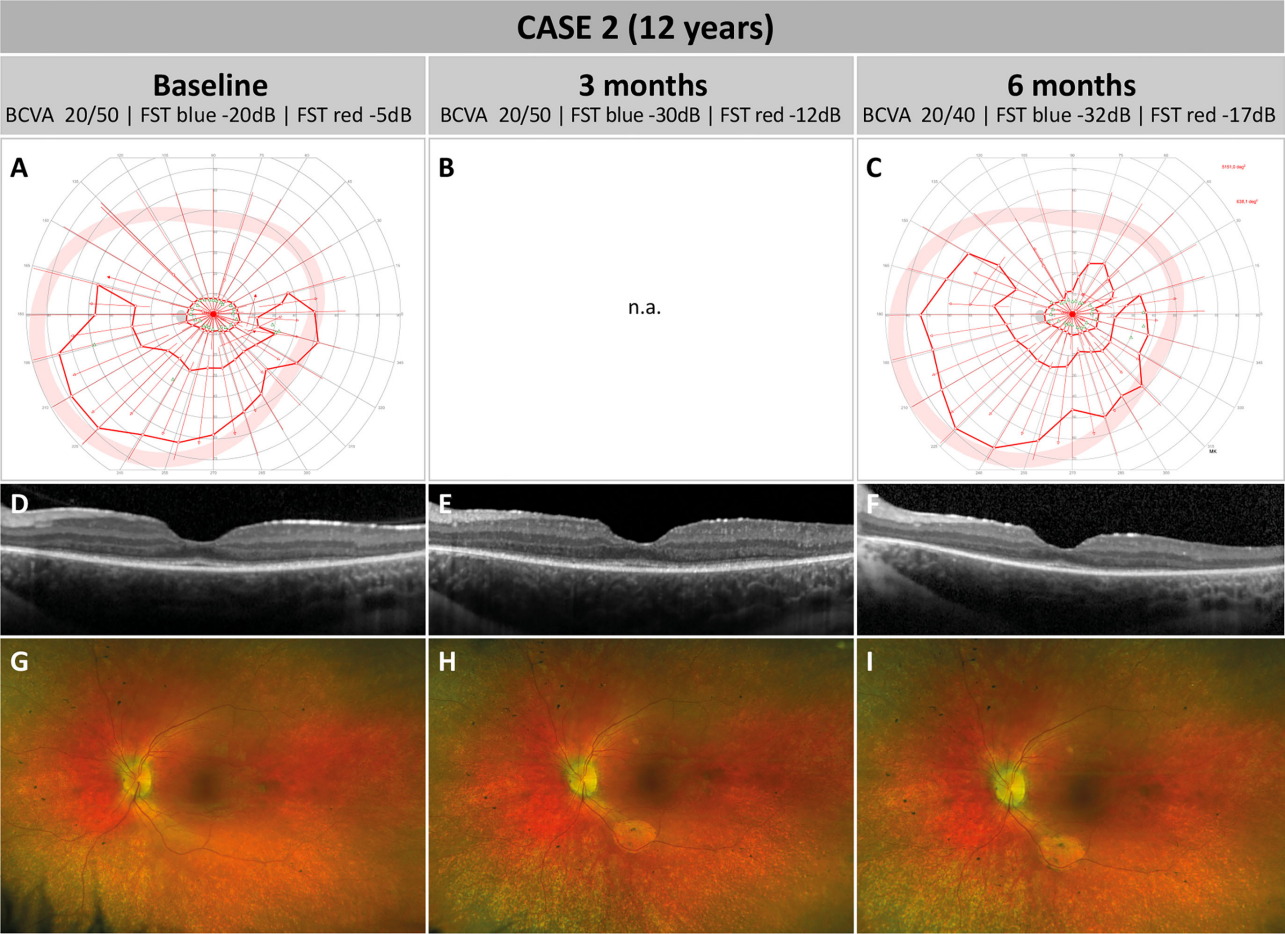
Pre- and post-treatment follow-up of case 2. (**A**–**C**) Left eye 90° kinetic perimetry using Goldmann object III4e. At baseline and 6 months after treatment, a remaining central visual field with concentric restriction to approximately 10° to 15° was observed with additional large inferior residual islands. At the 3-month follow-up, no kinetic perimetry was performed. No clinically relevant differences were observed between baseline and the 6-month follow-up. (**D**–**F**) Macular OCT scans of the left eye at baseline (**D**), 3 months after surgery (**E**), and 6 months after surgery (**F**). The residual island remained stable at 3 months after surgery but showed a slight reduction at 6 months. (**G**–**I**) CFP at baseline (**G**), 3 months after surgery (**H**), and 6 months after surgery (**I**). All pictures show a diffuse retinal atrophy typical of *RPE65*-IRD. At 3 months post-surgery, a newly developed area of CRA was observed at the lower vascular arcade and a smaller one around the upper vascular arcades, corresponding to the retinotomy sites. However, these areas remained stable (**H**, **I**).

During a follow-up examination 3 years later (baseline), at the age of 12, the girl presented with stable BCVA of 20/40 in the right and 20/50 in the left eye. However, her visual fields on 90° kinetic perimetry showed deterioration with complete ring scotomas and inferior residual islands in both eyes (Figs. 2C, 2D). FST measurements had worsened to −20 dB (−4 lg cd/m^2^) and −5 dB (−2.5 lg cd/m^2^) for blue- and red-light testing, respectively. As expected for *RPE65*-IRD, FAF images showed a reduction of overall signal intensity in both eyes (Figs. 2E, 2F), and CFP showed diffuse retinal atrophy without spicular intraretinal pigment (Fig. 3G). Macular OCT scans revealed bilateral parafoveal atrophy of the outer retinal layers with rather small preserved residual foveal islands (Fig. 3D; only left eye is shown). Faced with disease progression along with the increased clinical experience with the treatment, the parents consented to voretigene neparvovec treatment of the left eye as the subjectively worse eye to be the first one to treat.

Similar to case 1, during vitrectomy the detachment of the posterior vitreous was difficult, and strong vitreomacular adhesion made an injection of a second bleb around the lower vascular arcade necessary. Again, the fovea did not completely detach. Three months postoperatively, localized CRA at the retinotomy site was observed (Figs. 3H, 3I). Beyond that, surgery and post-operative management went as planned.

**Figure 4.**
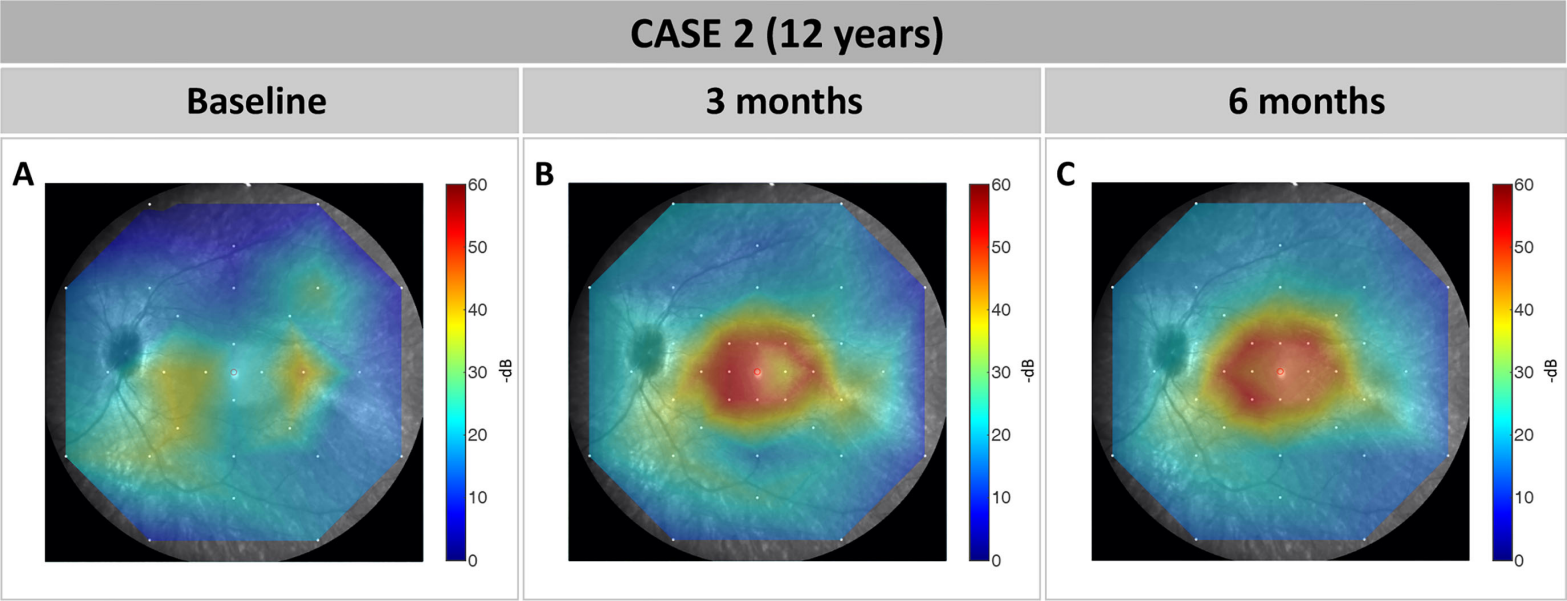
DACP for case 2. (**A**–**C**) Local dark-adapted thresholds using cyan stimulus with the DACP were overlaid on the fundus image of the patient at baseline (**A**), 3 months after surgery (**B**), and 6 months after surgery (**C**) for better visibility. The color-coding scale shows the local values in decibels (for comparison, a healthy eye has values around 60 dB in all locations). An improvement in local dark adaptation thresholds in the treated region is obvious and remained relatively stable in both postoperative visits. Of note, at baseline a shorter protocol with fewer stimulus locations designed specifically for children was used, whereas at the follow-up visits the patient was able to follow a standard protocol.

At the 3-month follow-up visit, BCVA of the treated left eye remained unchanged at 20/50 and showed a slight increase to 20/40 at 6 months, which was considered clinically insignificant. Fundoscopy and CFP at 3 and 6 months showed similar results compared to baseline, with the exception of CRA at the retinotomy sites around the upper and lower vascular arcades, which showed no enlargement over time (Figs. 3G–I). Similar to case 1, the FST values in the treated left eye improved to −30 dB (−5 lg cd/m^2^) and −12 dB (−3.2 lg cd/m^2^) at 3 months and to −32 dB (−5.2 lg cd/m^2^) and −17 dB (−3.7 lg cd/m^2^) at 6 months for blue and red light, respectively, showing a rod rescue effect. The configuration and area of the visual field remained stable (Figs. 3A–C). No relevant differences in the macular OCT scans were observed, with the size of the residual island remaining unchanged compared to baseline (Figs. 3D–F). Neither the patient nor the parents noticed any relevant changes in daily life after treatment. Intriguingly, DACP revealed an improvement of the local rod dark-adapted sensitivity located precisely to the treated macular area at 3-month and 6-month follow-up compared to baseline (Fig. 4).

### Case 3

A 12-year-old girl, diagnosed with *RPE65*-related early-onset retinal dystrophy (EORD), was referred to Ghent University Hospital at the age of 4 years. The main symptoms included reduced BCVA with infantile nystagmus, night blindness, and loss of midperipheral sensitivity of kinetic visual fields. Molecular genetic testing revealed two pathogenic variants (ACMG class 5) in *RPE65*: c.11+5G>A and c.991_993dupTGG(p.Trp331dup). Compound heterozygosity for the variants was confirmed through parental segregation analysis. The parents and patient agreed to bilateral treatment with voretigene neparvovec.

**Figure 5.**
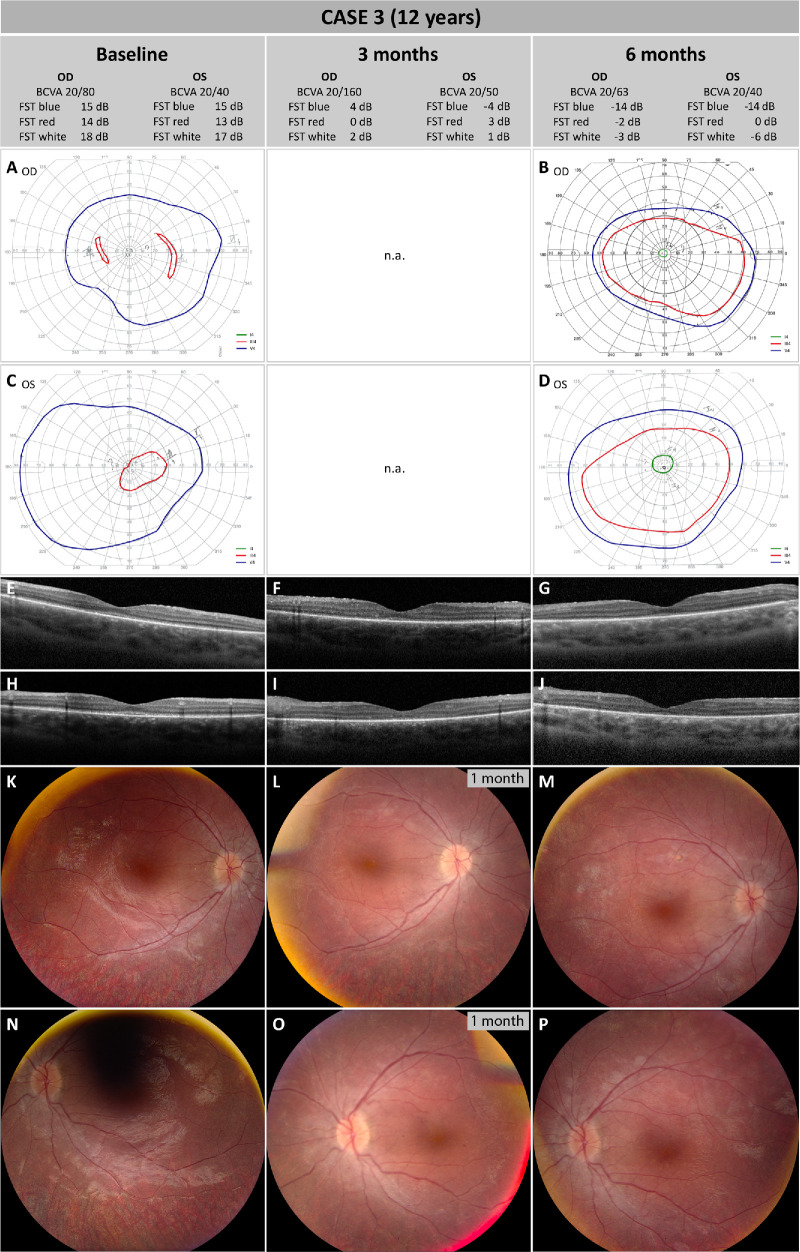
Pre- and post-treatment follow-up for case 3. (**A**–**D**) Right eye (**A**, **B**) and left eye (**C**, **D**) 90° kinetic perimetry using Goldmann objects V4e, III4e, and I4e. No kinetic perimetry was scheduled for the 3-month follow-up. No clinically relevant differences were observed between baseline and the 6-month follow-up for objects V4e and I4e; however, an improvement of visual fields using Goldmann object III4e was observed in both eyes. (**E**–**G**) Macular OCT scans of the right eye at baseline (**E**), 3-month follow-up (**F**), and 6-month follow-up (**G**). (**H**–**J**) Macular OCT scans of the left eye at baseline (**H**), 3-month follow-up (**I**) and 6-month follow-up (**J**). The residual island remained stable over time, and no other clinically relevant alterations were evident in both eyes. (**K**–**M**) CFP of the right eye at baseline (**K**), 1 month after surgery (**L**), and 6 months after surgery (**M**). No clinically relevant differences were observed between baseline and 6-month follow-up, except for the development of a small atrophic area within the vascular arcades at the retinotomy site superior to the macula. (**N**–**P**) CFP of the left eye at baseline (**N**), 1 month after surgery (**O**), and 6 months after surgery (**P**). No CFP images are available for the 3-month follow-up; instead, images from the 1-month follow-up are shown. All CFP images show a diffuse retinal atrophy typical for *RPE65*-associated IRD. No clinically relevant differences were observed between baseline and the 6-month follow-up.

At initial presentation (baseline), BCVA was measured at 20/80 and 20/40 for the right and left eye, respectively. Kinetic visual field testing of the right eye using object III4e showed only small paracentral residual islands with loss of central vision. Testing with V4e revealed only a mild constriction of the peripheral limits without additional scotomas. In the left eye, III4e showed a severe concentric constriction but a relatively well-preserved visual field with V4e (Figs. 5A, 5C). Light sensitivity measurements were severely abnormal at −0.56, −0.54, and −0.25 lg cd/m^2^ (14.4, 14.6, and 17.5 dB) for the right and −0.70, −0.48, and −0.33 lg cd/m^2^ (13, 15.2, and 16.7 dB) for the left eye for red, blue, and white light, respectively.

During surgery, detachment of the posterior vitreous was performed up to the retinal mid-periphery in both eyes. A single retinotomy was performed slightly superior and inferior to the superotemporal vascular arcades in the right and left eyes, respectively. The bleb included the fovea in both eyes. Postoperative management was favorable in both eyes.

At 3-month follow-up, BCVA was 20/160 in the right eye and 20/50 in the left eye and further improved to 20/63 in the right eye and 20/40 in the left eye at 6-month follow-up. In both eyes, a significant improvement was seen for light sensitivity at −1.96, −2.43, and −1.85 lg cd/m^2^ (0.4, 4.3, and 1.5 dB) for the right eye and −1.75, −2.35, and −1.86 lg cd/m^2^ (2.5, −3.5, and 1.4 dB) for the left eye for red, blue, and white light, respectively. At the 6-month follow-up visit, FST results further improved and measured −2.15, −3.41 and −2.33 lg cd/m^2^ (−1.5, −14, and −3.3 dB) for the right eye and −2.00, −3.41, and −2.60 lg cd/m^2^ (0, −14.1, and −6 dB) for the left eye for red, blue, and white light, respectively. No clinically relevant changes were observed for visual fields using objects V4e and I4e, although results for object III4e improved in both eyes (Figs. 5B–D).

CFP and FAF images are not available for the 3-month follow-up; therefore, images taken at 1 month post-treatment are shown. Fundoscopy and CFP at 1 and 6 months after treatment showed similar results compared to baseline in both eyes, except development of a small atrophic area within the vascular arcades at the retinotomy site in the right eye (Figs. 5H–M). No relevant differences in the macular OCT scans were observed, with the size of the residual island remaining unchanged compared to baseline (Figs. 5E–J). In addition to a globally reduced FAF signal intensity, a central macular ring with stronger autofluorescence compared to the surrounding retinal areas was seen on blue light FAF, with little change after treatment in both eyes (images not shown). After treatment, both parents noticed a change in visual function, mainly in dim light conditions, although subjectively this change was not experienced by the patient.

### Case 4

A 6-year-old girl was first referred to the Ghent University Hospital at 2.5 years of age due to night blindness that was first noticed at the age of 1 year. She was diagnosed with EORD, and genetic analysis revealed two heterozygous pathogenic (ACMG class 4) missense variants in *RPE65*: c.271C>T, p.(Arg91Trp) and c.1103A>G, p.(Tyr368Cys). Four more heterozygous variants were identified in *IQCB1*, *PDE6B*, *CDHR1*, and *CHM*, all found to be variants of unknown significance (ACMG class 3). Compound heterozygosity of the variants in *RPE65* was confirmed via parental segregation analysis. The main symptoms before treatment with voretigene neparvovec were night blindness and bumping into things when walking unaided in dimly lit environments.

**Figure 6.**
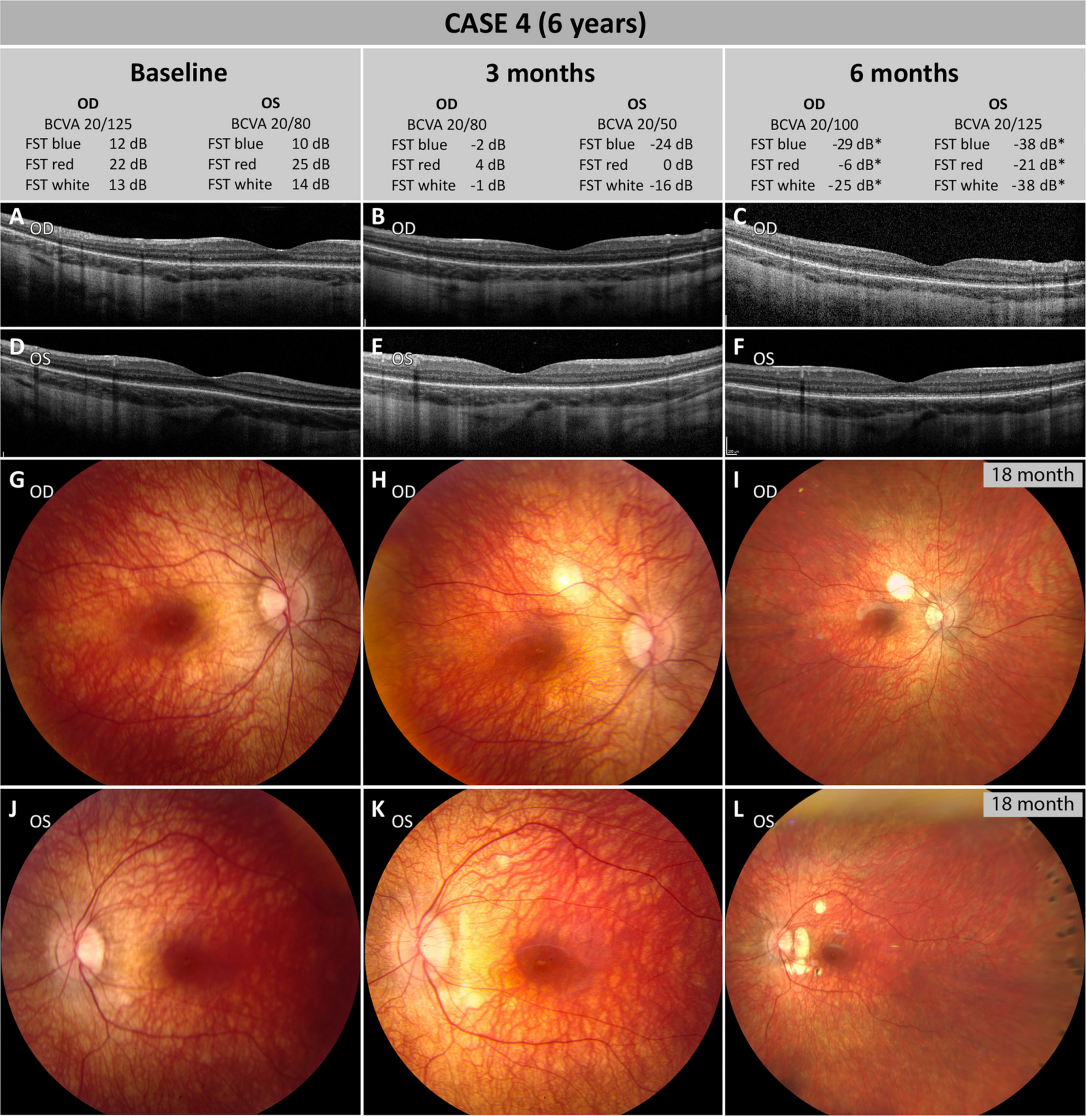
Pre- and post-treatment follow-up for case 4. (**A**–**C**) Macular OCT scans of the right eye at baseline (**A**), 3-month follow-up (**B**), and 6-month follow-up (**C**). (**D**–**F**) Macular OCT scans of the left eye at baseline (**D**), 3-month follow-up (**E**), and 6-month follow-up (**F**). The residual island remained stable over time, and no other clinically relevant alterations were evident in both eyes. (**G**–**I**) CFP of the right eye at baseline (**G**), 3 months after surgery (**H**), and 18 months after surgery (**I**). After surgery, a small area of CRA developed at the injection site at the superior vascular arcade. (**J**–**L**) CFP of the left eye at baseline (**J**), 3 months after surgery (**K**), and 18 months after surgery (**L**). After surgery, a small area of CRA developed at the injection site at the superior vascular arcade and small atrophic spots emerged temporally to the optic disc, which since have coalesced into a small area of CRA but remained stable until the last follow-up. No CFP images are available for the 6-month follow-up; instead, images from the 18-month follow-up are shown. *FST measurements at 6 months were unreliable; therefore, 12-month values are shown instead.

At baseline, the girl presented with a BCVA of 20/125 and 20/80 (measured using tumbling E charts) in the right eye and left eye, respectively. Light sensitivity results were severely anomalous at 0.15, −0.81, and −0.70 lg cd/m^2^ (21.5, 11.9, and 13 dB) for the right eye and −0.49, −1.05, and −0.59 lg cd/m^2^ (24.9, 9.5 and 14.1 dB) for the left eye for red, blue, and white light, respectively. Due to the girl's young age, visual field testing could not be performed. As this patient is an extremely shy, albeit intelligent girl, it is likely that all test results are an underestimation of her visual function. Macular OCT scans revealed parafoveal atrophy of the outer retinal layers with rather small preserved residual islands in the foveal area in both eyes (Figs. 6A–F). FAF imaging of the macula region showed globally diminished signal intensity as typically observed with *RPE65*-associated IRDs (images not shown). At this point, a joint decision with the parents was made to treat both eyes with voretigene neparvovec.

**Figure 7.**
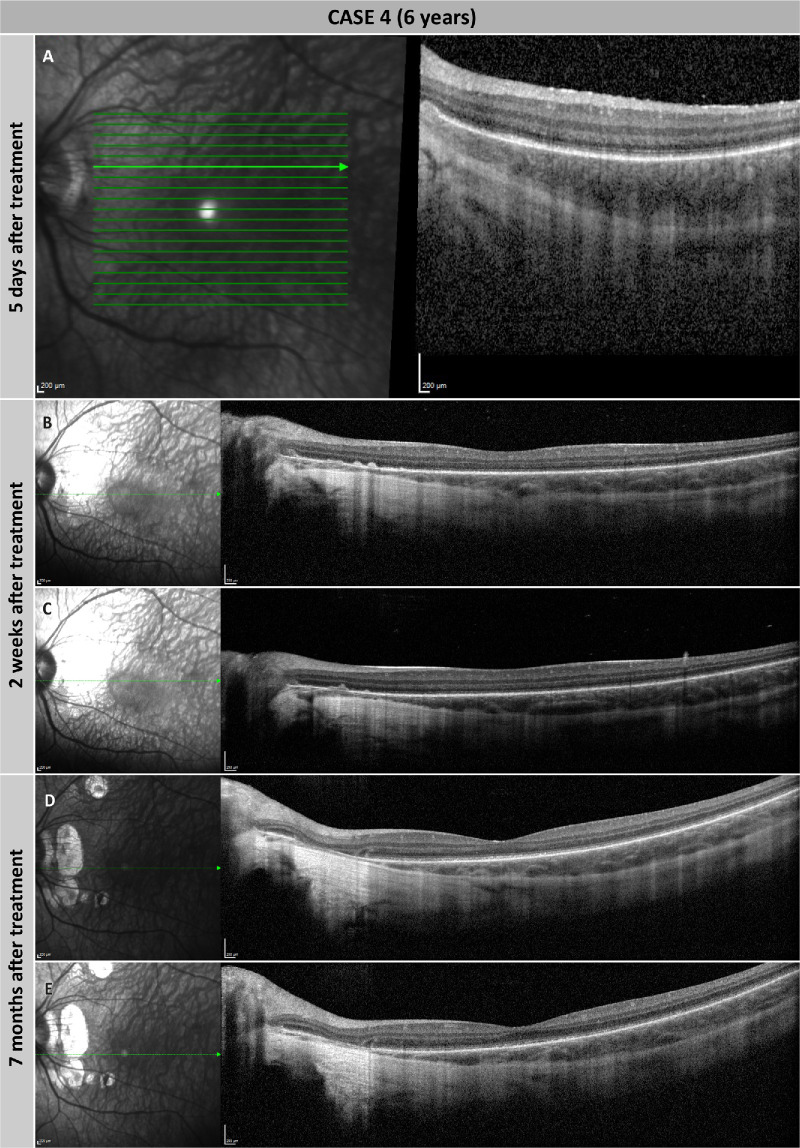
Subretinal deposits and development of CRA in case 4. (**A**) Macular OCT scan of the left eye 5 days after treatment. A small subretinal deposit near the optic disc and a decrease in choroidal thickness can be seen. (**B**, **C**) Follow-up examination at 2 weeks after treatment. Two distinct B-scans are shown, and both scans show enlargement of subretinal deposits. (**D**, **E**) Follow-up examination at 7 months after treatment. Both B-scans show extensive CRA in the area corresponding to the previously observed subretinal deposits.

During surgery, detachment of the posterior vitreous was performed up to the retinal mid-periphery in both eyes. A single retinotomy was performed around the superotemporal vascular arcades, and the subretinal bleb included the fovea in both eyes. Postoperative recovery after both treatments was uneventful, and the patient gained significant visual function bilaterally. However, 3 weeks after surgery spots of CRA were detected at the injection sites in both eyes and around the optic disc in the left eye (Figs. 6G–L). The areas of CRA have since remained stable in both eyes, and no negative impact on the visual function gained has been noted. Subretinal deposits, thought to be inflammatory cells, were seen prior to atrophy development in the postoperative period (Fig. 7).

At the 3-month follow-up, BCVA improved to 20/80 and 20/50 in the right eye and left eye, respectively. FST assessment showed significant improvement in both eyes at −1.58, −3.89, and −2.98 lg cd/m^2^ (4.2, −1.89, and −0.89 dB) for the right eye and −2.01, −4.41, and −3.61 lg cd/m^2^ (0.1, −24.1, and −16.1 dB) for the left eye for red, blue, and white light, respectively. At 6 months, BCVA was measured at 20/100 and 20/125 for the right eye and left eye, respectively. Due to the very limited patient cooperation during the 6-month follow-up, the BCVA values were deemed unreliable and did not align with the patient's and parents’ subjective descriptions. Likewise, the FST results with white light in the right eye and all colors in the left eye were unreliable at the 6-month follow-up time point (data not shown); therefore, light sensitivity results 12 months after treatment are reported. These results showed further improvement, reaching −2.59, −4.87, and −4.54 lg cd/m^2^ (−5.9 dB, −28.7 dB, and −25.4 dB) for the right eye and −4.13, −5.77, and −5.8 lg cd/m^2^ (−21.3 dB, −37.7 dB, and −38 dB) for the left eye for red, blue, and white light, respectively, demonstrating rod functional rescue. The absolute values are probably not entirely reliable due to limited patient cooperation, but the results show a clear tendency. Light sensitivity improved significantly in both eyes after treatment, and the strongest improvement was seen for light sensitivity for blue light, which likely represents an improvement in rod function. CFP images are not available for the 6-month follow-up; therefore, CFP images at 18 months are shown (Fig. 6). The macular OCT images showed no significant change of the outer retinal layers in the central macula in both eyes. Blue light FAF of the macula revealed stable results (data not shown).

## Discussion

Gene therapy in ophthalmology has undergone a revolutionary transition from an era when IRDs were deemed untreatable to a time when tangible therapeutic options are emerging. Historically, IRDs such as Leber congenital amaurosis and retinitis pigmentosa were considered incurable, casting a shadow of inevitability over progressive vision loss. The advent of voretigene neparvovec, the first approved gene therapy for *RPE65*-associated IRDs, heralded a new epoch in ophthalmology and has given hope to patients suffering from these devastating conditions. Despite it being a groundbreaking achievement, our experience with voretigene neparvovec is still limited, particularly regarding long-term outcomes and the unclear long-term development of treatment-associated fast-growing CRA.^13^ Treating advanced or fast-progressing cases, where blindness is a likely near-term scenario, is a relatively easy decision, but mild cases with well-preserved visual function require close evaluation and balancing of potential risks and benefits. Faced with the risk of losing visual function in a still relatively well-seeing eye, it is not surprising that many ophthalmologists refrain from treating very early and wait until relevant disease progression occurs. Additionally, the lack of a universally accepted definition of a mild phenotype further complicates decision-making. Without a clear framework, the variability in presenting symptoms and progression rates in mild cases makes it challenging to develop standardized treatment protocols. This lack of consensus often leads to more conservative approaches, delaying treatment until more definitive signs of disease progression are evident. However, the presence of a higher number of functional cells at the time of treatment significantly enhances therapeutic efficacy.^11^^,^^15^ We should therefore aim to treat as early as possible. Unfortunately, mild cases are rare and information on treatment outcomes is not readily available. In addition, molecular genetic evidence may not always be unequivocal due to the presence of rare or unique missense variants, with limited information on their true pathogenic impact. Hence, these are therefore sometimes deemed as variants of uncertain significance, as exemplified in case 2.

In this report, we present four patients with mild *RPE65*-related retinal disease phenotypes who received treatment with voretigene neparvovec in Germany and Belgium. The positive outcome of the *RPE65*-gene augmentation treatment on visual function in these patients further supports the pathogenicity of the four *RPE65* missense variants c.200T>A; p.(Leu67Gln), c.272G>A; p.(Arg91Gln), c.560G>A; p.(Gly187Glu), and c.1004A>G; p.(Glu335Gly). It is important to acknowledge that the two centers employed different examination protocols, which may complicate comparisons between cases. This is particularly relevant for dark-adapted threshold measurements. However, the primary aim of this study was to report individual patient outcomes rather than to make direct comparisons between the centers. In both German cases, we were hesitant to initiate treatment at first presentation, primarily due to comparatively well-preserved visual function and the unclear future rate of progression, the limited knowledge about treatment outcomes in the first few years after approval, and also because of the low variant classification in case 2. Both cases showed clear worsening over time, and in the face of progression it was decided to treat. It remains unclear how the disease would have progressed if we chose to treat earlier; however, based on the available evidence and our own experience, we might assume that long-term treatment efficacy would have been better, as more preserved retinal cells would have been available. In the context of surgical risks, especially at an early age, the risk of developing post-treatment CRA and knowledge of the benefits of early treatment, we find ourselves in a situation where we must balance the potential loss of treatment efficacy against the risks of treatment-associated side effects. Ultimately, the complexity and multifaceted nature of such cases highlight the need for a multimodal diagnostic approach to make informed treatment decisions.

We are currently at a stage where more information on treatment outcomes in mild cases is much needed. Hence, every IRD expert with similar cases should be encouraged to make information on treatment outcomes and individual disease courses publicly available. Although the cases presented herein show proven progression, they provide some insights into outcomes of treating relatively mild cases at a pediatric age. All four children benefited from the treatment and none of them experienced relevant surgical complications, despite issues due to subretinal adhesions in the German cases. Despite the relatively short observation period of 6 months, we did not observe the perimacular growth of fast-growing CRA that is usually seen from around month 3 of the postsurgical period. Only non-progressive CRA was detected at the retinotomy sites in three cases. This observation also supports the metabolic hypothesis of the development of CRA, which assumes that a large rescue effect in a retinal degeneration already showing progression might lead to a metabolic overload in the retinal tissue, with subsequent atrophic sequelae.

The additional CRA seen on the temporal side of the optic disc within the bleb area in case 4 may have, at least in part, been due to insufficiently controlled subretinal inflammation. Indeed, the subretinal deposits seen on OCT (Fig. 7) in the weeks prior to the appearance of the CRA are likely subretinal inflammatory cells. As these were not considered as such at the time, the immunosuppressive regimen was not adapted. Ghent University Hospital has since updated the procedures to include rapid commencement of higher dosed systemic and/or local steroids during a prolonged period, with progressive tapering dependent upon disappearance of subretinal deposits.

Early treatment in a situation without profound retinal degeneration, as was the case in these children, may have been the correct choice, also in view of preventing the fast-growing post-treatment development of CRA. Given the positive results, early treatment seems to be beneficial, even though predictions are very difficult in individual cases of mildly affected children. In this light, the parents of both German children opted for treatment of the second eye. Of note, other *RPE65*-IRD phenotypes that exhibit little or no progression, akin to those associated with congenital stationary night blindness, may warrant a more conservative approach, as we do not know whether treatment would provide benefits in these scenarios.
